# Biotechnological Applications of Biogenic Nanomaterials from Red Seaweed: A Systematic Review (2014–2024)

**DOI:** 10.3390/ijms26094275

**Published:** 2025-04-30

**Authors:** Aline Nunes, Graziano Rilievo, Massimiliano Magro, Marcelo Maraschin, Fabio Vianello, Giuseppina Pace Pereira Lima

**Affiliations:** 1 Department of Chemical and Biological Sciences, Institute of Biosciences, São Paulo State University, Botucatu 18618-970, São Paulo, Brazil; alinenunes_bio@hotmail.com (A.N.); pace.lima@unesp.br (G.P.P.L.); 2 Department of Comparative Biomedicine and Food Science, University of Padova, Viale dell’Università 16, 35020 Legnaro, Padua, Italy; graziano.rilievo@unipd.it (G.R.); fabio.vianello@unipd.it (F.V.); 3 Department of Plant Science, Federal University of Santa Catarina, Florianópolis 88034-000, Santa Catarina, Brazil; m2@cca.ufsc.br

**Keywords:** Rhodophyta, crops, nanotechnology, therapeutics, bio-coronas

## Abstract

Green synthesized nanoparticles (NPs) are arousing constantly increasing attention due to inherent advantages such as biocompatibility, nontoxicity, and cost-effectiveness. As the state of the art of this rapidly evolving topic demands a punctual update, the present study was focused on reviewing the novelty, feasibility, and effectiveness related to the specific category of red seaweed-derived NPs. Among algae, red seaweeds have already gained consideration in the global market due to their high content of primary and secondary metabolites, supporting multifunctional applications across various industries. This scoping review reveals how this interest has also driven their investigation as a natural source for the sustainable NP fabrication. The fragmentary body of studies was synthesized, identifying red seaweed NPs as a flourishing nanotechnological subgroup and meriting their own space in the scientific literature. Noteworthy, the great majority of the reviewed papers feature efficient controlled release, enhanced bioavailability, and reduced toxicity, making red seaweed NPs elective candidates for the medical sector as anticancer, antimicrobial, and antioxidant agents. Moreover, their parent natural counterparts seem to endow NPs with unexpected specificity toward biological targets such as prokaryotic and tumor cells. Nanotechnological solutions based on red seaweeds pave the way to a new avenue of opportunities and challenges.

## 1. Introduction

From industry to several scientific branches, nanomaterials are appealing for their advantageous distinctive characteristics [1,2]. However, conventional synthetic routes involve expensive chemical and/or physical processes that are a matter of environmental concern, in particular in view of large-scale production of nanoparticles (NPs) [3]. Indeed, the employment of harmful chemicals such as reducing agents, surface stabilizers, and organic solvents poses a potential obstacle to NP implementation in several fields [4].

Green synthesis represents a highly attractive alternative to these industrial-scale NP processes since it employs natural materials that are characterized by huge availability, non-toxicity, and biodegradability [5]. Furthermore, besides being beneficial to the environment, green synthesis is also cost-effective and easy to execute, whereas traditional approaches often involve high energy consumption and the management of toxic by-products downstream of the synthetic process [4].

Among natural sources, seaweeds have become an increasingly important focus for the global market, particularly due to their richness in primary metabolites (amino acids, sugars, fatty acids, polysaccharides, organic acids, proteins, fibers, vitamins, and biogenic amines) and secondary metabolites (phenolic compounds and pigments) [6,7]. This abundance has led to advancements for various multifunctional applications across multiple industries, including pharmaceuticals/medicine, agriculture, food, bioremediation, biofuels, and cosmetics/dermatology [8]. More specifically, these plant secondary metabolites can serve as reducing and capping agents, promoting the bottom-up conversion of metal ions into NPs while effectively inhibiting their aggregation [5].

Among categories of seaweeds classified by their pigments, i.e., brown algae (Ochrophyta, Phaeophyceae), green algae (Chlorophyta), and red algae (Rhodophyta), the latter stands out, particularly for their composition in fatty acids, pigments, polyphenols, and polysaccharides, which can provide health benefits and can be utilized as ingredients for innovative formulations [9]. The versatility of this group allows their application across various industries, primarily due to the presence of polysaccharides, such as carrageenans and agars (i.e., sulfated galactans) [10].

The global market of red algae is projected to exceed 3.2 billion USD by 2032, with a compound annual growth rate (CAGR) of 4.6% from 2022 to 2032. This growth is primarily driven by the increasing demand for natural active ingredients in personal care and cosmetic products. In addition to regions with well-established cultivations, such as China and Indonesia, the market is expected to witness growth in other countries [11].

Red seaweeds are one of the most diverse and ecologically significant algal groups, characterized by their unique pigmentation and complex life cycles [10]. They are widely distributed in aquatic environments and have been extensively studied for their rich composition of bioactive compounds, including amino acids, fatty acids, vitamins, minerals, phenolic compounds, pigments such as phycoerythrin, and carbohydrates [12]. These compounds contribute to a variety of biological activities, including antioxidative, antimicrobial, anti-inflammatory, and anticancer effects, making red algae particularly appealing for applications in the pharmaceutical, agricultural, and industrial fields [10,13,14]. A study by Yun et al. [15] investigated the effects of sugars derived from agar extracted from red algae, finding that agarotriose exhibited in vitro prebiotic activity, while 3,6-anhydro-l-galactose significantly inhibited the proliferation of human colon cancer cells and induced apoptosis. Aluta et al. [16] demonstrated that sulfated polysaccharides from *Centroceras clavulatum* displayed functional properties, including immunostimulatory and antioxidant activities. The predominant compound, galactose, has potential nutraceutical applications due to its ability to stimulate carp leukocyte culture cells, enhancing intracellular antioxidant defenses at relatively low concentrations. Sharan and Vennila [17] reported that extracts from *Kappaphycus alvarezii* exhibited strong antioxidant activity and potential efficacy against oral pathogenic microbes, paving the way for developing dental products to combat oral infections. Additionally, Sudhakar, Dharani, and Paramasivam [18] found that extracting and purifying the pigment protein phycoerythrin from *Gracilaria corticata* resulted in significant total antioxidant activity, effective in DPPH scavenging and iron ion chelation. In other *Gracilaria* species, such as *G. edulis* and *G. salicornia*, Vasudevan et al. [19] identified 15 compounds capable of crossing the blood-brain barrier out of 48 and 86 total compounds, respectively. The ethanolic and methanolic extracts of these two species demonstrated mild antiproliferative properties in glioblastoma cell lines, suggesting therapeutic benefits. Thus, red algae merit attention not only for their ecological relevance but also for their biotechnological potential, justifying the growing scientific interest in this group [10,13,14].

Relatively few examples of the use of seaweeds as nanomaterial synthetic precursors can be found in the scientific literature prior to 2010 [20]. Differently, in the years comprised between 2010 and 2013, albeit the topic was still in its infancy, the future blooming of green-synthesized nanoparticles from algae can be disclosed by the emergence of novel studies, already putting red seaweeds in the limelight. This prompt interest was clearly driven by the aforementioned biological properties along with the general attractiveness of nanomaterial green synthesis [21] and the demand for alternatives for waste recycling [22]. These early publications already revealed some important clues about the influence of the parent algal matrix on the NP bio-corona compositions. Indeed, the FTIR profile of biogenic silver nanoparticles from *Gelidiella acerosa* showed towering amide I and II bands, substantiating the formation of an amino acid-rich shell [21]. The bio-reduction of gold ions using *Chondrus crispus* extract as a reducing-capping agent was accompanied by the formation of a biocorona composed of proteins and xanthates [22]. Green synthesized Ag nanoparticles from *Gracilaria corticata* displayed prominent FTIR peaks ascribed to phenolic compounds, amide I groups, and aromatic rings involved in the stabilization of the final Ag nanomaterial [23]. Among other components, it is worth mentioning the presence of phenolic compounds, which could endow the nanomaterial with antimicrobial, anticarcinogenic, and antioxidant properties [24]. Indeed, the nanomaterial surface, facing the milieu, provides the biological identity and activity to the abiotic core. Actually, the biomolecular corona on nanoparticles influences the fate of the nano-bio-conjugate in targeting the biological recipients, and this drastically depends on the different repertoire of biomolecules enriching the nanomaterial’s outermost shell [25].

Overall, the variety of bioactive compounds present in different red seaweeds can likely lead to a plethora of possible bio-corona combinations and, therefore, to a number of unpredictable and possibly advantageous interplays with prokaryotic and eukaryotic cells.

As an example, the potential of red seaweed-derived nanoparticles as anticancer therapeutics was already highlighted in 2014, successfully demonstrating the efficiency of gold and silver nanoparticles from *Corallina officinalis* and *Pterocladiella capillacea*, respectively [26,27]. Not surprisingly, the decade encompassed by the current review was characterized by a constantly increasing number of publications, in particular in the pharmaceutical and biomedical fields.

Considering the specificities of algal compounds and the increasing market demand, research on algal-derived nanoparticles (NPs) has grown significantly. This increase is attributed to the unique properties of proposed NPs, which can allow the controlled release of drugs, enhanced bioavailability and effectiveness, and reduce toxicity, among other beneficial aspects [28]. Nevertheless, it should be pointed out that reproducibility is a crucial scientific tenet, in particular, for the biomedical field [29]. The richness of bioactive molecules in algal extracts represents a double-edged sword, counterpoising to the aforementioned potential benefits the risk of a lack of reproducibility concerning the actual composition of the synthetic products and, as a consequence, their bioactivity. Indeed, it should be recalled that the mechanism of action of isolated algal bioactive species (vide supra) is far from being comparable to their nano-immobilized counterparts, in particular if the chemical nature of the bio-corona remains obscure. However, this represents a general issue for all biogenic nanomaterials and probably the main bottleneck hampering their widespread use and their scale-up at an industrial level of production [30]. Thus, in order to take full advantage of these promising biogenic nanomaterials, the correlation between their biological activities and the exact bio-corona compositions needs to be addressed. It is of pivotal importance to fill the gap existing between our knowledge about the composition and the activity of a natural matrix and the control over a designed core-shell nanomaterial fabrication. Anyway, in light of this emerging scenario, the present study aims at exploring the properties and applications of nanoparticles derived from red algae, analyzing their efficacy and their potential for innovation across various industrial sectors. Overall, the pursuit of sustainable and effective solutions, combined with the growing interest in the properties of seaweeds, opens up a range of opportunities for the research and development of new products, which can meet the demands of the contemporary market.

## 2. Results

### 2.1. Paper Selection and Characteristics

**Figure 1:**
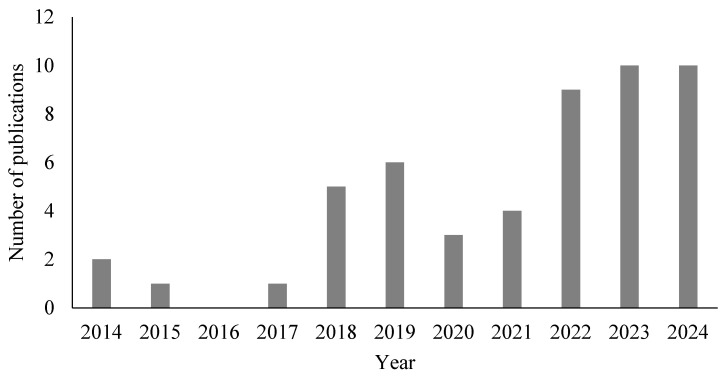
Number of publications per year (2014–2024) using the descriptors “red seaweed” and “nanoparticles” in the PubMed and Web of Science databases.

Among the 49 papers selected with the defined inclusion criteria, it was found that the highest publication rates were in 2023 (n = 9) and in 2024 (n = 9). In 2022 (n = 9), followed by 2019 (n = 6), 2018 (n = 5), 2021 (n = 4), 2020 (n = 3), 2014 (n = 2), 2015 (n = 1), 2017 (n = 1), and 2016 (n = 0) (Figure 1). Thus, it is evident that publications in recent years (2022–2024) were prominent, and this may be attributed to the increasing interest in utilizing red macroalgae for producing novel NPs.

Regarding journals involved in the publication’s occurrence, the most notable were the International Journal of Biological Macromolecules (n = 4), Process Biochemistry (n = 3), and Marine Drugs, Nanomaterials, Particulate Science and Technology, and Asian Pacific Journal of Cancer Prevention (n = 2 each). The remaining journals (n = 34) published only one article on the subject.

Regarding algal genera used for the development of NPs, a total of 24 genera were identified, with Gracilaria being the most prominent, featuring 11 distinct studies. Two studies were reported using the genera Acanthophora, Gelidium, Halymenia, Hypnea, Palmaria, Porphyra, and Spyridia, while the remaining genera (Calliblepharis, Champia, Chondrus, Corallina, Crassiphycus, Galaxaura, Gelidiella, Grateloupia, Jania, Kappaphycus, Laurencia, Laurenciella, Porphyridium, Pterocladia, Pterocladiella, and Solieria) involved only one research paper. Thus, among the species of red algae used in the reported studies, 39 were described, namely *Acanthophora spicifera*, *Acanthophora* sp., *Calliblepharis fimbriata*, *Champia parvula*, *Chondrus crispus*, *Corallina officinalis*, *Crassiphycus birdiae*, *Galaxaura oblongata*, *Gelidiella acerosa*, *Gelidium amansi*, *Gelidium corneum*, *Gracilaria birdiae*, *Gracilaria canaliculata*, *Gracilaria corticata*, *Gracilaria crassa*, *Gracilaria debilis*, *Gracilaria edulis*, *Gracilaria firma*, *Gracilaria fisheri*, *Gracilaria foliifera*, *Gracilaria lemaneiformis*, *Gracilaria verrucosa*, *Grateloupia lithophila*, *Halymenia porphyriformis*, *Halymenia venusta*, *Hypnea musciformis*, *Hypnea valentiae*, *Jania rubens*, *Kappaphycus alvarezii*, *Laurencia aldingensis*, *Laurenciella* sp., *Palmaria decipiens*, *Palmaria palmata*, *Porphyra linearis*, *Porphyra* sp., *Porphyridium cruentum*, *Pterocladia capillacea*, *Pterocladiella capillacea*, *Solieria robusta*, *Spyridia filamentosa*, and *Spyridia hypnoides*.

**Figure 2:**
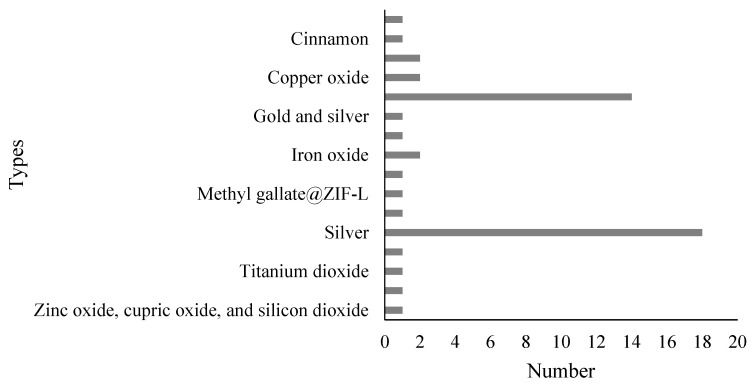
Basis for the formulation of nanoparticles using red seaweed (2014–2024).

Various materials were utilized as a basis for the development of NPs, highlighting silver (n = 18) and gold (n = 14), followed by copper, copper oxide, and iron oxide (n = 2 each). Chitosan-tripolyphosphate, cinnamon, gold with silver (mix), graphene oxide, metformin-ferrous-ferric oxide, methyl gallate@zif-l, selenium, titanium dioxide, silver with zinc oxide (mix), zinc oxide, and finally, the mix of zinc oxide, cupric oxide, and silicon dioxide were tested once (Figure 2).

In the present work, three categories were created to describe the results, considering the 49 published manuscripts, with one of them presenting results from two areas. The pharmaceutical area represented 79% of the studies, making it the focus of the research. However, the other areas were also addressed, as they represent a gap in research in recent years: general industry (n = 8) and agriculture (n = 3).

### 2.2. Pharmaceutical/Medical Field

A total of 39 papers were identified in the pharmaceutical/medical field, published in the following years: 2014 (n = 2), 2016 (n = 1), 2017 (n = 1), 2018 (n = 4), 2019 (n = 5), 2020 (n = 3), 2021 (n = 2), 2022 (n = 6), 2023 (n = 6), and 2024 (n = 9). The investigated biological activities included: anticancer/cytotoxic/antitumoral activity (n = 21), antimicrobial activity (n = 17), antioxidant activity (n = 11), larvicidal activity (n = 2), drug monitoring and detection (n = 2), as well as specific actions, such as biocompatibility (n = 1), anticoagulant (n = 1), anti-inflammatory (n = 1), neurotoxicity (n = 1), hypoglycemic (n = 1), hemolysis (n = 1), prediction of renal lesions (n = 1), and imaging agents in drug delivery (n = 1). The total number of biological activities studied (n = 59) exceeded the number of articles (n = 39), indicating that several articles analyzed more than one biological activity (Table 1).

Regarding the algal species studied, the actions of natural products from *Hypnea valentiae* (n = 3), *Kappaphycus alvarezii* (n = 3), *Champia parvula* (n = 2), *Gracilaria verrucosa* (n = 2), and *Pterocladiella capillacea* (n = 2) were discussed. This was followed by *Acanthophora* sp., *Acanthophora spicifera*, *Chondrus crispus*, *Corallina officinalis*, *Crassiphycus birdiae*, *Gelidium amansii*, *G. corneum*, *Gelidiella acerosa*, *Gracilaria birdiae*, *G. corticata*, *G. debilis*, *G. firma*, *G. fisheri*, *G. foliifera*, *G. lemaneiformis*, *G. oblongata*, *Halymenia porphyriformis*, *Halymenia venusta*, *Jania rubens*, *Laurencia aldingensis*, *Laurencia* sp., *Palmaria decipiens*, *Porphyra linearis*, *Porphyridium purpureum*, *Solieria robusta*, and *Spyridia filamentosa*, each mentioned only once. In addition to these species, carrageenan (either commercial or obtained from a research institution) was utilized (n = 3), as well as extracts derived from the Porphyra genus (commercial form—nori) (n = 2).

Studies on the algal extract *H. valentiae* used it as an encapsulating agent for silver NPs (AgNPs), Viswanathan et al. [31] developed a green methodology for producing 10–45 nm-sized spherical NPs. The biomedical potential of these NPs was evaluated by antioxidant, antibacterial, and anticancer assays. The DPPH assay showed a strong ability of AgNPs to eliminate free radicals. Moreover, AgNPs demonstrated significant antibacterial activity against *Pseudomonas aeruginosa*, *Enterococcus faecalis*, *S. aureus,* and *Streptococcus mutans*. Notably, AgNPs were particularly effective against *E. faecalis* and *S. mutans*, outperforming controls (i.e., algal extract, silver nitrate, and ampicillin). Regarding the Minimum Inhibitory Concentration (MIC), AgNPs matched ampicillin only for *S. mutans* at 12.5 µg/mL, although higher doses (25–100 µg/mL) were needed to compare the antibiotic (6.25–25 µg/mL) on other microorganisms. In anticancer tests, AgNPs showed excellent activity against HT-29 colon cancer cells (IC_50_ = 24.6 μg/mL) and A549 lung cancer cells (IC_50_ = 5.9 µg/mL) by the MTT assay. Therefore, these NPs are promising for medical applications, exhibiting antibacterial, antioxidant, and anticancer properties. In a subsequent study by Viswanathan et al. [32] using the same algal species (*H. valentiae*), the anticancer and antioxidant activities were evaluated on gold NPs (AuNPs) prepared by the seaweed. The AuNPs ranged in size from 7 to 45 nm and exhibited a spherical shape. The antioxidant assays demonstrated significant free radical-scavenging capabilities by DPPH, hydrogen peroxide, and FRAP assays at an AuNPs concentration of 50 μg/mL for all experiments. The anticancer assessment was performed on A549 lung cancer cells, yielding an IC_50_ of 23.68 μg/mL. These findings indicated that metal nanoparticles coated by *H. valentiae* possess excellent antioxidant and anticancer activity.

Baskar et al. [33] also studied *H. valentiae* for the eco-friendly production of iron oxide NPs (IONPs), which were in the size range from 20 to 40 nm and were tested for the DPPH antioxidant and anticancer activity against lung cancer (A549) and breast cancer (MDA-MB-231) cell lines. According to antioxidant assays, free radical elimination varied with dosage, achieving a maximum inhibition of 87.52% at a concentration of 100 µg/mL, with an IC_50_ at 55.24 µg/mL. Regarding anticancer efficacy, the inhibition fraction of both cancer cell types increased with NP dosage, and the half-maximal inhibitory concentration for A549 cells was 66.79 µg/mL, while for MDA-MB-231 cells it was approximately 33.89 µg/mL, demonstrating the effectiveness of these IONPs at lower doses against breast cancer cells compared to lung cancer cells. For this reason, these NPs could be further explored for innovative pharmaceutical products tailored for breast cancer treatment.

In a paper investigating the possible use of *K. alvarezii*, Khan, Ranjani, and Hemalatha [34] described the synthesis of silver NPs using an extract from the macroalgae and their antibacterial activity against five strains of *E. coli* (i.e., EC ATCC 25922, EC 13, EC 36, EC 3T, and EC 16). The average size of these AgNPs was found to be 80 nm, and an inhibition activity was demonstrated on four *E. coli* strains (i.e., EC 13, EC ATCC 25922, EC 16, and EC 3T), indicating good antibacterial activity. The inhibiting capacity was evaluated by the Agar well diffusion method, with 25 µg/mL and 50 µg/mL AgNPs. For the EC 13 strain, the inhibition zone was 21 mm at 25 µg/mL and 22 mm at 50 µg/mL; for the EC ATCC 25922 strain, the inhibition zone was 17 mm and 19 mm at 25 and 50 µg/mL, respectively; for the EC 16 strain, it was 9 mm at both concentrations; and for the EC 3T strain, it was 10 mm at both concentrations. Therefore, these AgNPs might be used to prevent bacterial infections from *E. coli* and could serve as a safe and effective alternative to antibiotics.

Jaffar et al. [35] reported on silver NPs synthesized using a green method using *K. alvarezii* as a reducing and stabilizing agent. The AgNPs exhibited cubic-shaped aggregates with rough surfaces composed of spherical nanoparticles, which measured 1–8 nm in diameter. The AgNPs were tested for antimicrobial activity against two Gram-positive bacteria (i.e., *Staphylococus aureus* and *Bacillus subtilis*), two Gram-negative bacteria (i.e., *E. coli* and *Klebsiella pneumoniae*), and the fungal species *Candida albicans*. Four NPs concentrations (0.1, 0.25, 0.5, and 1 mg/mL) were tested, revealing that *B. subtilis* and *E. coli* showed the highest susceptibility to AgNPs compared to *K. pneumoniae* and *S. aureus*. The MIC was consistently observed at 0.13 mg/mL for all tested microorganisms. In terms of minimum bactericidal concentration (MBC), both *S. aureus* and *B. subtilis* displayed MBC values equal to their MIC (0.13 mg/mL), suggesting that AgNPs are not only inhibitory but also bactericidal. Higher concentrations (0.25 mg/mL) were required for *E. coli*, *K. pneumoniae*, and *C. albicans* for a bactericidal effect. Thus, the green-synthesized silver NPs confirmed their significant antibacterial action, paving the way for a promising avenue for biomedical applications.

A new multifunctional hybrid system for drug delivery was developed by Kesavan et al. [36]: Chitosan (CS)-functionalized graphene oxide (GO) capped with the polysaccharide xyloglucan (XG), named GO-CS-XG. The ternary hybrid was employed as a nanocarrier for κ-carrageenan extracted from *K. alvarezii* (GO-CS-XG-κ-C), which served as an anticancer drug. The GO-CS-XG-κ-C had a size of 270 nm and was tested in vitro on human breast cancer (MCF-7) and human lymphoma (U-937) cell lines. After a 48 h treatment at 100 μg/mL nanohybrid, the U-937 cell viability assessed by MTT assay for GO-CS-XG was 82.54%, while for GO-CS-XG-κ-C it was 35.05%. On the other hand, cell viability for MCF-7 was 55.98% for the GO-CS-XG treatment and 24.23% for the GO-CS-XG-κ-C treatment. In addition, an effective pH stimuli-responsive κ-carrageenan release from the nanocarrier was successfully achieved for the first time. GO-CS-XG loaded with κ-carrageenan exhibited high cytotoxic activity for killing cancer cells; therefore, the authors suggested its application as a biocompatible nanomaterial as an anticancer drug.

Regarding studies utilizing *Champia parvula*, two papers were found, both from the same research group [37,38]. Viswanathan et al. [37] described the biogenic synthesis of gold NPs (AuNPs) and evaluated their antioxidant and anticarcinogenic activity. The spherical NPs were obtained with a size of 20 nm and exhibited significant antioxidant potential, with increased free radical scavenging activity as the NP concentration increased. At a concentration of 50 mg/mL, the highest inhibition recorded by the DPPH assay was 80.2%, followed by 74.7% by the H_2_O_2_ test and 85.2% by the FRAP assay. In terms of anticarcinogenic activity, it was observed that on lung cancer cell lines (A549), cell viability gradually decreased from 3 to 50 mg/mL AuNP. The lowest cell viability (46.7%) was found at a concentration of 50 mg/mL, with an IC_50_ value of 36.08 mg/mL, confirming that these AuNPs could be further evaluated for the development of new nano-anticancer drugs.

Again, Viswanathan et al. [38] developed silver NPs (AgNPs) using *C. parvula*, evaluating the antioxidant, antimicrobial, and anticancer properties. The AgNPs exhibited a spherical morphology and a size of 79 nm. Antioxidant analysis showed that the fraction of free radical inhibition increased with AgNP concentration. At a concentration of 50 μg/mL, the DPPH assay exhibited an inhibition of 91.34%, while the H_2_O_2_ test showed a 76.4% inhibition and the FRAP assay a 75.6% inhibition. In terms of antimicrobial activity, the AgNPs significantly controlled the growth of bacterial pathogens, such as *S. mutans*, *S. aureus*, and *E. faecalis*, as well as the fungal species *C. albicans*. At a concentration of 100 μL/mL, the AgNPs showed appreciable inhibition of *S. mutans* (23 mm), *S. aureus* (21 mm), *E. faecalis* (20 mm), and *C. albicans* (20 mm) by the Agar well diffusion method, although the control (i.e., streptomycin at 30 μL/mL) demonstrated a larger inhibition zone. Anticancer activity of AgNPs against human lung cancer (A549) and colon cancer (HT-29) cell lines was tested. The growth inhibition gradually increased with AgNP concentration, and the IC_50_ was found to be 21.54 μg/mL for A549 cells, while the IC_50_ for HT-29 cells was approximately 42.36 μg/mL, indicating that A549 cells treated with AgNPs demonstrated a higher sensitivity compared to HT-29 cells. Thus, the study contributed to the recent view of developing new biogenic nanodrugs for cancer and microbial infections.

Chellapandian et al. [39] described multifaceted gold NPs obtained by a green synthesis involving *G. verrucosa*. These AuNPs, presenting both isotropic and anisotropic forms with sizes ranging from 20 to 80 nm, were tested for biocompatibility against normal human embryonic kidney (HEK-293) cell lines. The AuNP concentrations tested (i.e., 0–100 μg/mL) showed an almost nil cytotoxic effect on the cells. A slight cell death (<10%) was observed after 24 h of AuNP exposure only at the highest concentration (i.e., 100 μg/mL) by the MTT viability assay, whereas, under the same conditions, the Trypan blue test showed a survival rate of 95%. Thus, these AuNPs can be considered for future applications as biocompatible nanocarriers.

Copper(II) oxide NPs (CuONPs) were developed by Marmiroli et al. [40], using *Gracilaria verrucosa* and *Ulva lactuca* to evaluate their antimicrobial properties. The CuONPs ranged in size from 30 to 40 nm, and their activity was analyzed against five microorganisms: *Saccharomyces cerevisiae*, *C. albicans*, *E. coli*, *B. subtilis*, and *S. aureus*. The CuONPs synthesized from macroalgae did not inhibit any of the microorganisms, except for *E. coli*, which was inhibited by the red algae-derived CuONPs at 500 mg/L (i.e., the highest concentration tested). For authors, this result might be related to nanoparticle aggregation, therefore affecting their bioavailability. Clearly, further studies on these copper oxide NPs obtained by green synthesis are needed.

El Kassas & Attia [27] evaluated the bactericidal application and anticancer activity of AgNPs formed by *Pterocladiella capillacea* extract. The AgNPs exhibited a spherical shape, with an average size of 11 nm, and were assessed for their cytotoxicity against human hepatocellular carcinoma cell lines (HepG2) and bactericidal activity against *S. aureus*, *B. subtilis*, *P. aeruginosa*, and *E. coli*. Results revealed that the IC_50_ for the HepG2 tumor cells was 3.7 µg/mL. Additionally, a significant direct dose-response relationship was observed, with increased cytotoxicity at higher concentrations. Moreover, the lowest concentration tested (0.3 µg/mL) was still able to inhibit cell growth. For bactericidal activity, a dose-dependent effect was also noted. At 20 µg/mL, the Agar well diffusion method showed inhibition zones of 25.1 mm for *B. subtilis* and 18.1 mm for *S. aureus*. In contrast, the activity against *P. aeruginosa* (11.1 mm) and *E. coli* (6.5 mm) was significantly lower, indicating a stronger effect of AgNPs against Gram-positive strains. Again, these NPs might be considered for new anticancer approaches and for bactericidal applications, especially against Gram-positive bacteria.

Two iron oxide NPs (IONPs)-based hybrids were obtained using the brown alga *Colpomenia sinuosa* and the red alga *P. capillacea* and were formulated by Salem, Ismail, & Aly-Eldeen [41], who evaluated their antimicrobial effects. The IONPs by *P. capillacea* exhibited a size of 16.9–22.5 nm and were tested against Gram-negative bacteria (*E. coli*, *P. aeruginosa*, *S. typhi*, and *Vibrio cholera*), Gram-positive bacteria (*B. subtilis* and *S. aureus*), and two pathogenic fungi (*Aspergillus flavus* and *Fusarium oxysporum*). Strong antibacterial activity was observed by the Agar well diffusion test, with the largest inhibition zone (15 mm) for *V. cholerae*, while *S. aureus* showed the least inhibition effect (3 mm) with *P. capillacea*-produced IONPs. Regarding the pathogenic fungi, a higher antifungal activity was achieved with the *C. sinuosa*-iron oxide NPs, inhibiting *A. flavus* (9 mm) and *F. oxysporum* (6 mm), compared to the other microorganisms. Thus, these magnetic nano-bio-conjugates can be considered promising candidates for pharmaceutical products.

The study by Krishnaswamy et al. [42] investigated the use of *Acanthophora* sp., demonstrating its applicability for the green synthesis of copper NPs. The obtained CuNPs, with a quite large size of 0.5 μm, exhibited a strong anticoagulant effect at 100 μg/mL in both the prothrombin time assay and the activated partial thromboplastin time assay, suggesting their potential as a naturally derived anticoagulant. Babu et al. [43], synthesizing gold NPs with *A. spicifera*, produced a nanomaterial with an average size below 20 nm and a spherical shape. These AuNPs were evaluated for antioxidant, antibacterial, and anticancer activities. They exhibited superior antioxidant activities compared to the extract of *A. spicifera* alone, showing the highest inhibitory activities against the DPPH radical (62.8%) and the NO_2_ radical (59.2%) at 500 µg/mL. Regarding their antibacterial activity, strong inhibition was observed against *Vibrio harveyi* (22 mm) and *S. aureus* (19 mm) when using 100 µg/mL AuNP in the Agar well diffusion test. Moreover, anticancer efficacy on the human colon adenocarcinoma cell line (HT-29) was used as a model, revealing an IC_50_ value of 21.86 µg/mL AuNPs.

All these NPs demonstrated a broad range of therapeutic properties, providing solid evidence for future research on the development of new biomedical drugs. González-Ballesteros et al. [44] reported on the green synthesis of AuNPs from three different red seaweed extracts: *C. crispus* (AuNP@CC), *G. corneum* (AuNP@GC), and *P. linearis* (AuNP@PL). The AuNP@CC and AuNP@PL nanoparticles exhibited an average diameter of 16.9 nm, while Au@GC measured 44.2 nm. Notably, *P. linearis* extract showed superior capacity to reduce Au(III) to Au(0) during the AuNP@PL production process. After the synthesis, the antioxidant, antitumor, and anti-inflammatory potentials of these AuNPs were evaluated. The antioxidant analysis using human promyelocytic cells (HL-60) showed that all samples reduced basal ROS release, except Au@CC, *C. crispus* extract, and *P. linearis* extract. In terms of DPPH scavenging activity, Au@PL demonstrated the highest antioxidant activity, capable of reducing ROS to baseline levels even at low concentrations. Moreover, two different cell lines were tested for antitumor activity of Au nanoparticles: lung epithelial tumor cells (A549) and monocytic tumor cells (THP-1). No significant differences were observed between A549 cells and untreated cells for any of the AuNPs or their corresponding extracts at all tested concentrations. However, AuNP@GC and AuNP@PL exhibited intrinsic immunomodulatory activity, which could be further explored for immunotherapeutic applications.

The cytotoxic activity of gold NPs synthesized with C. officinalis extract on the cancer cell line MCF-7. The AuNPs, with an average diameter of 14.6 nm, exhibited remarkable cytotoxic effects against MCF-7 cells, causing necrosis at concentrations of 3 and 6 µg/mL, with an IC_50_ of 1.5 μg/mL. Thus, these NPs showed potential as antitumor agents for new approaches in cancer therapy [26].

A green self-assembly of silver NPs using sulfated polysaccharides from the seaweed *C. birdiae* was proposed by Medeiros et al. [45]. The polysaccharides-derived NPs exhibited an average size of 117.6 nm, and the assessed antioxidant activity resulted in 10% higher than controls (unmodified AgNPs).

Pugazhendhi et al. [46] synthesized silver NPs (AgNPs) using the seaweed *Gelidium amansii* and tested their antimicrobial activity against various pathogenic bacteria (i.e., Gram-positive bacteria: *S. aureus* and *Bacilus pumilus*; Gram-negative bacteria: *E. coli*, *P. aeruginosa*, *V. parahaemolyticus*, and *Aeromonas hydrophila*). The AgNPs exhibited a spherical shape, with sizes ranging from 27 to 54 nm, and showed a 99% reduction of biofilm formation. Significant results were observed for all bacteria strains, especially against *P. aeruginosa*, *V. parahaemolyticus*, and *S. aureus*. Thus, *G. amansii*-sourced NPs might be used as coating agents for various surfaces to control bacterial biofouling.

The synthesis of gold NPs (AuNPs) using an extract of *G. acerosa* resulted in AuNPs with diameters ranging from 5 to 20 nm and a spherical shape [47]. The AuNPs were then tested for antibacterial activity against *S. aureus* with different treatment concentrations (i.e., 0, 25, 50, 75, and 100 µg/mL) in disc diffusion assays. A decrease in the bacterial colony patches was observed, showing cell death in a dose-dependent response, with the largest inhibition zone at 100 µg/mL. These AuNPs might serve as potential candidates for biomedical applications for antibacterial activities.

In the study by Aragão et al. [48], the green synthesis of silver NPs was explored using polysaccharides extracted from *G. birdiae*, and their antibacterial activity against *E. coli* and *S. aureus* was evaluated. The hydrodynamic diameter of these AgNPs ranged from 20.2 nm to 94.9 nm, depending on the polysaccharide concentration used during the green synthesis (i.e., 0.02–0.05%) and the pH conditions (i.e., pH 10 or pH 11). For 0.05% polysaccharide-synthetized AgNPs at pH 11, significant activity was observed, particularly against *E. coli*, with an MIC of 81.2 μg/mL. These results further suggested that AgNPs synthesized by red algae extracts can be used as a model for future nanomedicine purposes.

A novel silver NPs using an extract from *G. corticata* (Gc-AgNPs) was developed by Naveenkumar et al. [49], and their larvicidal and neurotoxic activities were investigated. The Gc-AgNPs were spherical and monodisperse, with an average size of 24 nm. In the larvicidal activity tests, the behavior of Gc-AgNPs was observed in fourth instar larvae of *Aedes aegypti*, *Anopheles stephensi*, and *Culex quinquefasciatus*, with 100% mortality observed after 48 h. The LC_50_ values were 17.0, 13.6, and 14.1 μg/mL, respectively. Moreover, larval acetylcholinesterase (AchE) activity was significantly suppressed upon exposure to Gc-AgNPs compared to the controls. Larvae of *A. stephensi* treated with Gc-AgNPs at a dose of 6.5 μg/mL exhibited a higher AchE activity than controls, followed by *C. quinquefasciatus* and *A. aegypti*. AchE activity progressively decreased in larvae at increasing concentrations of Gc-AgNPs. Additionally, a toxicity test conducted on the freshwater crustacean Daphnia magna revealed that the neonates became immobile after exposure to Gc-AgNPs with doses ranging from 6.25 to 100 μg/mL for 24 and 48 h, leading authors to affirm that Gc-AgNPs are less toxic and biocompatible on non-target species. These results suggested that silver NPs synthesized using algae extracts could serve as potential nano-larvicidal agents.

Prabhu et al. [50] described a one-pot fabrication of nanocomposites formed by zeolitic imidazole frameworks (ZIF-L) encapsulated in methyl gallate (MG@ZIF-L) *from G. debilis*. The average particle size of the MG@ZIF-L was 217 nm. After characterization, MG@ZIF-L was evaluated for its anticancer potential against A549 lung cancer cell lines by MTT and LDH assays. The treatment of cells with MG@ZIF-L exhibited significant cytotoxic effects in a dose-dependent manner compared to MG alone. According to the MTT assay, the IC_50_ value was 72.56 μg/mL. Furthermore, an acute toxicity assay was conducted using zebrafish embryos as an alternative method. In these experiments, embryos treated with MG@ZIF-L showed no significant malformations even at the highest dose tested (200 μg/mL). Therefore, this zeolitic nanocomposite might be considered as a promising agent for lung cancer treatment.

Kalimuthu et al. [51] developed novel silver NPs by a green synthesis using different concentrations of aqueous extract of *G. firma*, and these AgNPs were tested on the predatory efficiency of the copepod *Megacyclops formosanus* and on the dengue vector, *A. aegypti*. The AgNPs exhibited a spherical shape, with 80% ranging from 12 to 200 nm in size. The larvicidal activity was tested on the first, second, third, and fourth instar larvae, as well as pupae of *A. aegypti*. The LC_50_ value of the *G. firma* extract against *A. aegypti* ranged from 0.091% (*v*/*v*) (first instar) to 2.4% (*v*/*v*) (pupae), while the LC_50_ of AgNPs varied from 26 mg/L (first instar) to 350 mg/L (pupae). However, when assessing the toxicity of the G. firma AgNPs on *M. formosanus*, mortality was recorded with an LC_50_ of 350 mg/L. In predation tests, younger instars (first and second) were found to be more susceptible than copepods. The authors suggested that AgNPs from *G. firma* present the potential to be used as a new and eco-friendly larvicidal agent against *A. aegypti*.

Kamble et al. [52] developed bimetallic NPs (BNPs) of gold and silver using sulfated galactan (SG) extracted from *G. fisheri*. These BNPs (SG-BNPs) were tested for antibacterial activity against shrimp pathogens *V. parahaemolyticus* (strains VH-3HP and VH-A3212) and *V. harveyi* (strains VH0-1114 and BAA-1116). The BNPs exhibited spherical morphology with a narrow size distribution of 3–10 nm. Inhibition zones in Agar plates against VH-3HP, VH-A3212, VH0-1114, and VHBAA-1116 in the presence of SG-BNPs (a 250 μg/mL SG and 13 μg/mL BNPs formulation) were 13, 13.5, 12, and 14 mm, respectively. For bare BNPs (13 μg/mL), the test showed inhibition zones measuring 9, 9.5, 10, and 11.5 mm, while pure SG (2 mg/mL) displayed only 7.0, 6.5, 6.5, and 6.5 mm for the three bacterial strains, respectively. Additionally, diets supplemented with SG-BNPs, BNPs, and SG significantly improved the survival rate of shrimp infected with *V. parahaemolyticus* (VH-3HP strain) compared to controls. Thus, SG-BNPs might serve as an aquaculture antibacterial tool to face Vibrio infections in shrimp, being non-toxic and environmentally friendly.

Various algae, green (*U. rigida*), brown (*Cystoseira myrica*), and red (*G. foliifera*), were studied by Algotiml et al. [53] as reducing and capping agents to synthesize gold NPs (AuNPs). The application of the red algae extract produced the largest nanoparticles, measuring 13 nm, compared to 9 nm obtained by the green and 11 nm by brown algae extracts. The cytotoxicity of nanoparticles was assessed on Hfb-4 skin cell lines, the anticancer activity on the MCF-7 cancer cell model, and antimicrobial properties were tested on bacteria (*B. cereus*, *S. aureus*, *E. coli*) and on fungi (*C. albicans*, *Trichosporon cataneum*, and *Trichophyton mentagrophytes*). Cytotoxicity of AuNPs on Hfb-4 cells showed a good dose-dependent response, with cell viability decreasing as AuNP concentration increased. Cells treated with AuNPs obtained with the *G. foliifera* extract showed a viability of 91.8% at the lowest concentration tested (0.092 µg/mL), dropping to 13.3% at the highest concentration (188 µg/mL). The IC_50_ of the red algae-capped AuNPs was 64 µg/mL, resulting in an intermediate value between brown (102 µg/mL) and green (32 µg/mL) algae-synthesized AuNPs. Anticancer assays on MCF-7 by AuNPs from *G. foliifera* exhibited the highest anticancer activity (92.13%) at 188 µg/mL, with an IC_50_ of 30 µg/mL. Regarding the antimicrobial activity, the green algae-obtained AuNPs showed superior effects compared to the two other formulations. Fungi, particularly *T. cataneum* and *T. mentagrophytes*, were very sensitive to AuNPs from all algae extracts. These AuNPs obtained by using different algal extracts hold promise as alternative anticancer and antimicrobial agents.

In the study by Tang et al. [54], selenium NPs (SeNPs) were developed using polysaccharides extracted from *G. lemaneiformis*, resulting in spherical particles approximately 90 nm in size. The analysis included antioxidant, hypoglycemic, cytotoxic, and hemolytic activities. The SeNPs formulated by using the algae extract showed superior performances compared to controls, achieving 103.4%, 94.2%, and 86% for DPPH, ABTS, and O_2_•^−^ radical scavenging ability, respectively, at a concentration of 1.5 mg/mL. Furthermore, these SeNPs exhibited high inhibitory effects on α-amylase and α-glucosidase, with IC_50_ values of 1.6 mg/mL and 2.1 mg/mL, respectively. Cytotoxicity of these SeNPs was assessed using a mouse macrophage cell line (RAW 264.7 cells), revealing a dose-dependent decrease in viability, ranging from 0.5 to 20 μg Se/mL, while cell viability remained around 77% at higher NP concentrations. Hemolysis rates were below 5% even at the highest concentration tested (i.e., 2 mg Se/mL), indicating a potential for pharmaceutical applications. For this reason, these SeNPs might be an interesting candidate for diabetes treatment and as an antioxidant supplement or ingredient.

The development of predictive algorithms for acute kidney injury (AKI) in hospitalized patients, by a machine learning approach, utilizing titanium dioxide and an extract from *G. oblongata*, which can be incorporated into various nanoparticle formulations, was explored by Tu et al. [55]. Among the predictive models, logistic regression exhibited lower overfitting compared to support vector machines and random forests, demonstrating its effectiveness in identifying AKI risk factors. Authors identified lymphocytes and myoglobin (≥1000 ng/mL) as independent risk parameters for AKI. Additionally, the GCS score served as a risk indicator for 60-day mortality, with a high 90-day mortality rate among AKI patients (*p* < 0.0001). Treatment with TiO_2_ NP was also linked to renal damage in experimental samples. The research highlighted that the marine extract of *G. oblongata* might protect against acute renal injury by reducing oxidative stress and inflammatory responses and decreasing serum cytokines, such as lactoperoxidase (LPO), nuclear factor kappa-light-chain-enhancer of activated B cells (NF-kB), and myeloperoxidase (MPO). According to the authors, the observed beneficial effects can be ascribed to the antioxidant activity of this extract, which can be ideally used for the fabrication of other nanoparticles with the aim of coping with inflammation and organ injury induced by LPS.

Khan et al. [56] synthesized silver NPs using extracts from *H. porphyriformis* and *S. robusta,* and the produced AgNPs were assessed for their efficacy against oral pathogenic bacteria (*S. aureus*, *Streptococcus viridans*, *Lactobacillus acidophilus*, and *Lactobacillus brevis*). The AgNPs from *H. porphyriformis* had comparable diameters to those from *S. robusta*, measuring 15 and 17 nm, respectively. Both AgNPs exhibited moderate antibacterial activity against all tested bacterial strains, except *L. acidophilus*. Under the experimental conditions, the best results of *H. porphyriformis*-synthesized AgNPs were an MBC of 50 μg/mL against *L. brevis*. For *S. robusta*, the MBC was 200 μg/mL against *S. aureus* and *S. viridans*, 100 μg/mL against *L. acidophilus*, and 50 μg/mL against *L. brevis*. Thus, these AgNPs could be further tested as antibacterial agents for treating dental bacterial pathogens.

The antioxidant, antimicrobial, and anticancer activities of Gold NPs synthesized from *H. venusta* were evaluated. The AuNPs were spherical, measuring 81 nm in size, and exhibited a strong inhibitory effect on DPPH free radicals, with a maximum inhibition of 64.4% at a concentration of 50 µg/mL. Regarding the antimicrobial activity against *S. aureus*, *P. aeruginosa*, and *Klebsiella pneumoniae*, the highest inhibition zone was observed against *P. aeruginosa* in a dose-dependent manner. In anticancer activity tests, growth inhibition of lung cancer cells (A549) occurred after 24 h, with an IC_50_ of 39.04 µg/mL [57].

The antioxidant activity of *J. rubens* extracts encapsulated in chitosan-sodium tripolyphosphate NPs (JCNPs) was measured by Maghraby et al. [58]. These nanoparticles, characterized by sizes ranging from 161 to 669 nm, were studied as a function of the concentration of chitosan and sodium tripolyphosphate. The antioxidant properties of these NPs were assessed, and due to the total phenolic content (TPC) of *J. rubens*, 121 mg GAE/g (i.e., mg of gallic acid equivalents per g dry weight), and the total flavonoid content (TFC), 60.9 mg QE/g (i.e., mg of quercetin equivalents per g of dry weight). DPPH and FRAP assays demonstrated that the extract exhibited radical scavenging activity ranging from 10.3% to 50.2%, depending on the concentration used, showing a linear increase with the JCNPs dose. These results suggest the potential use of these nanoparticles due to their strong antioxidant activity for various applications.

Vieira et al. [59] developed silver NPs using aqueous extracts of *L. aldingensis* and *Laurenciella* sp., assessing their cytotoxic and anticancer activities. The silver nanoparticles exhibited hexagonal and triangular shapes with an average size of 100 nm. Cytotoxicity assays were conducted on P4 cells (human prepuce fibroblasts) and cancerous MES-SA/Dx5 cells (human uterine sarcoma with doxorubicin resistance) alongside their parental MES-SA lineage. Authors reported that for healthy human P4 cells, cell viability was approximately 70% after 24 h of incubation at an NP concentration of 3.0 μM, indicating low toxicity. However, on MES-SA and MES-SA/Dx5 sarcoma cells, high toxicity was observed, with cell viability dropping to 3% for MES-SA and 12% for MES-SA/Dx5 at 5 μM AgNPs. On MES-SA, the IC_50_ values were 1.07 μM for *L. aldingensis* and 0.97 μM for *Laurenciella* sp.-derived AgNPs. Regarding experiments on MES-SA/Dx5 cells, AgNPs from *L. aldingensis* and *Laurenciella* sp. showed IC_50_ values of 1.68 and 1.62 μM, respectively. Thus, these AgNPs demonstrate significant cytotoxicity against sarcoma tumor cells.

A core-shell nanobiocomposite was synthesized using R-phycocyanin pigment extracted from *P. purpureum* as a coverage for a bimetallic core composed of silver and zinc oxide (Ag-ZnO). The average size of the as-obtained core-shell hybrid was 33 nm. The nanomaterial showed a dose-dependent cytotoxicity against the MCF-7 cell line [60]. Cell viability decreased with nanocomposite concentration, with a maximum cytotoxicity of 59%. The IC_50_ value was determined at an NP concentration of 100 μg/mL. This evidence could represent a significant contribution to the field of cancer therapy.

In the study by Valarmathi et al. [61], the biosynthesis of silver NPs using the extract from *S. filamentosa* was described, assessing their antibacterial properties against *Klebsiella* sp. and *Staphylococcus* sp., as well as their cytotoxic effects on MCF-7 cells. The AgNPs exhibited a spherical structure with a size ranging from 20 to 30 nm. According to the authors, the antibacterial activity showed a dose-dependent response, with a concentration of 20 mM AgNPs inhibiting Klebsiella sp. growth by 63.4% over 48 h, while the inhibition against *Staphylococcus* sp. was only 44.6% under the same conditions. Cytotoxicity results indicated that cell viability decreased with increasing concentrations of AgNPs (25, 50, 75, and 100 µg/mL), demonstrating an inverse relationship between the amount of AgNPs and cell survival. Thus, as the authors pointed out, these red algae-derived NPs might serve as an effective antibacterial agent in medical applications.

In the study conducted by González-Ballesteros et al. [62], *Palmaria decipiens* was evaluated for its potential as a reducing and stabilizing agent in the green synthesis of nanoparticles (NPs). Aqueous extracts from this macroalga were utilized to synthesize gold and silver NPs, resulting in particles with diameters of 37 nm and 7 nm, respectively. Fourier Transform Infrared Spectroscopy (FTIR) analysis of the nanomaterials indicated the presence of functional groups linked to proteins, phenols, and polysaccharides, which may play a crucial role in the reduction and stabilization of the gold and silver NPs. The plasmon resonance properties of these nanoparticles suggest promising applications in the biomedical field, including use as imaging agents in drug delivery, gene therapy, and thermotherapy.

In addition to the aforementioned manuscripts, other articles utilized specifically purified carrageenan [63,64], as well as commercially obtained carrageenan [65] and commercially obtained porphyran from the genus Porphyra [66,67], both extracted from red algae. Chen et al. [63] synthesized gold NPs using carrageenan oligosaccharides (CAO) with an average diameter of 35 nm. After characterization, these AuNPs were tested for antitumor activity against human breast cancer cells (MDA-MB-231), human colon cancer cells (HCT-116), and human umbilical vein endothelial cells (HUVEC). The CAO-derived AuNPs exhibited significant cytotoxic activity against HCT-116 and MDA-MB-231 cells, with IC_50_ values of 34.4 μg/mL and 129.2 μg/mL, respectively, after 72 h of incubation. Thus, these AuNPs might serve as a promising nanomaterial for anticancer applications.

In another study by Chen et al. [64], the same CAO-AuNPs were proposed as a delivery system for epirubicin (EPI). The EPI-CAO-AuNPs were spherical and monodisperse, displaying an average size of 141 nm. HepG2 and HCT-116 (i.e., cancer cell lines) and HUVEC cells (i.e., human normal cells) were tested, showing that CAO-AuNPs had good biocompatibility, with over 95% cell viability in normal cells. Moreover, EPI-CAO-AuNPs reduced the cytotoxicity effect of soluble EPI in normal cells. On the contrary, EPI-CAO-AuNPs showed a dose-dependent reduction of HepG2 cell viability, registering a significantly stronger effect than the soluble EPI used as a control. Indeed, after 72 h, the IC_50_ values were 0.087 and 0.173 μM for EPI-CAO-AuNPs and EPI, respectively. The nanomaterial could therefore merit being considered for targeted drug delivery in cancer therapy.

Nanorods consisting of copper oxide (CuO) functionalized with gallic acid (Ga@CuO) and loaded with paclitaxel (PTX) were developed by Singh and Pal [65]. To control PTX release, nanorods were coated with κ-carrageenan and further functionalized with folic acid (FA) as a cancer cell-targeting biomolecule. The as-obtained Ga@CuO-PTX@K-carrageenan and Ga@CuO-PTX@K-carrageenan/FA nanocomplexes had a size of approximately 170 nm and 190 nm, respectively. Cytotoxicity tests showed strong effects (IC_50_ 12 μg/mL) and a high fraction of apoptotic cells (40.25%) in the MCF-7 cell line, indicating the effective PTX release from the nanosystem. Additionally, changes in cancer cell metabolic activity and inhibition of cell proliferation were interpreted as apoptosis features induced by reactive oxygen species (ROS). Treatment with Ga@CuO-PTX@K-carrageenan/FA also reduced mitochondrial membrane potential, leading as well to cell apoptosis. This work further promotes the novel strategy of targeted drug delivery by applying tailored inorganic-organic nanocomplexes involving algal biomolecules.

Lima et al. [66] described the use of carbon paste electrodes modified with gold NPs coated with porphyran (PFR, a sulfated polysaccharide obtained from red algae) (CPE/AuNPs-PFR) for detecting the anticancer drug 5-fluorouracil (5-FU). The smallest particle size achieved was 129 nm. The CPE/AuNps-PFR exhibited high sensitivity to 5-FU, with a limit of detection (LOD) of 0.66 μM and a limit of quantification (LOQ) of 2.22 μM. In addition, the modified carbon paste electrode was successfully applied for drug detection in an injectable pharmaceutical sample, representing a viable alternative for electrochemical sensing of 5-fluorouracil (5-FU) in pharmaceutical formulations.

Similarly, Bojko et al. [67] synthesized silver NPs using the same polysaccharide, porphyran, as a coating agent and D-glucose as a reducing agent. The particles had an average diameter of 24 nm and were tested for antibacterial activity against *S. aureus* and *E. coli*. The antibacterial activity showed inhibition zones for both species at a concentration of 55 μg/mL nanocomplex for sterile paper disks, with *E. coli* exhibiting a 1.9 cm inhibition zone and *S. aureus* a 2.3 cm inhibition zone. This nanomaterial was also used as a modifier of carbon paste electrodes for the electrochemical sensing of 5-FU, displaying detection and quantification limits of 10.7 μM and 35.8 μM, respectively. This envisions the potential of the nanomaterial as a promising analytical detection tool for control of the anticancer drug.

**Table 1:** Published articles in the PubMed and Web of Science databases (2014–2024) dealing with nanoparticles produced with red seaweed components for pharmaceutical/medical applications.

Nº	Target Effect	Species Used	Core Nanoparticle	Results/Potential	References
1	Cytotoxic activity in cancer cell lines	*Corallina officinalis*	Gold	Strong NP cytotoxic activity was observed against breast cancer cells, indicating its potential for anticancer therapy	[26]
2	Bactericidal application and cytotoxic activity	*Pterocladiella capillacea*	Silver	Strong anticancer activity and high bactericidal efficacy of NPs exhibited against Gram-positive strains, highlighting their potential in cancer treatment and bacterial infections	[27]
3	Cytotoxic activities	*Laurencia aldingensis* and *Laurenciella* sp.	Silver	The NPs demonstrated low toxicity to healthy human cells and high cytotoxicity against uterine sarcoma cells, highlighting their potential as therapeutic agents for tumor treatment	[59]
4	Vector control	*Gracilaria firma*	Silver	Significant larvicidal activity of NPs was observed, proving their application as a potential eco-friendly larvicidal agent against *A. aegypti*	[51]
5	Antitumor activity	In-house purified carrageenan	Gold	NPs exhibited significant cytotoxic activity against cancer cells, highlighting their potential for the formulation of cancer treatments	[63]
6	Methodology optimization	*Palmaria decipiens*	Gold and silver	The green synthesis led to NPs with potential therapeutic and medical applications due to the active components involved in the reduction and stabilization processes	[62]
7	Monitoring 5-fluorouracile in pharmaceutical formulations	Porphyra genus (commercial form—nori)	Gold	The proposed device demonstrated high sensitivity to 5-fluorouracile and was successfully applied for the analysis of injectable pharmaceutical samples, as a viable alternative for drug monitoring in pharmaceutical formulations	[66]
8	Antimicrobial activity	*Gelidium amansii*	Silver	Strong antimicrobial activity was observed, indicating the potential of AgNPs as a coating material for biofouling control	[46]
9	Antibacterial activity	*Gracilaria birdiae*	Silver	Antibacterial activity of AgNPs against *E. coli* and *S. aureus* was observed, suggesting that these NPs can be used as a model for future nanomedicine projects or drug delivery systems	[48]
10	Biocompatibility against different cell lines	*Gracilaria verrucosa*	Gold	These AuNPs demonstrated high biocompatibility on normal cells, showing no significant cytotoxic effects, and suitable are for future applications as drug nanovehicles	[39]
11	Anticancer drug release	In-house purified Carrageenan	Gold	These AuNPs were developed as a drug delivery system showing higher biocompatibility on normal cells than soluble drug (epirubicin) and stronger dose-dependent cytotoxicity against HepG2 cells, suggesting their potential as targeted drug delivery agent	[54]
12	Anticancer activities	*Gracilaria debilis*	Methyl gallate encapsulated on ZIF-L	Significant cytotoxic effects were observed against lung cancer cell lines, suggesting its application as a promising agent for cancer treatment	[50]
13	Antimicrobial activity	*Pterocladiella capillacea*	Iron oxide	These IONPs exhibited a strong antibacterial activity and a low antifungal activity, suggesting their use for pharmaceutical and biomedical applications, particularly as antibacterial agent	[41]
14	Antioxidant, antibacterial, and anticancer activities	*Acanthophora spicifera*	Gold	Significant antioxidant, antibacterial, and anticancer activities of these AuNPs were observed, highlighting their potential for the development of new drugs	[43]
15	Antibacterial activity e detection of 5-fluorouracil (5-FU)	Porphyra genus (commercial form—nori)	Silver	AgNPs exhibited antibacterial activity against *S. aureus* and *E. coli* and were applied for the electrochemical detection of 5-FU, demonstrating the potential as a promising analytical tool for quality control	[67]
16	Antibacterial and anticancer activity	*Spyridia filamentosa*	Silver	Dose-dependent antibacterial activity and cytotoxic effects of AgNPs on breast cancer cells were obtained, highlighting their potential for applications in food, textile industries, and medicine	[61]
17	Antioxidant activity	*Crassiphycus birdiae*	Silver	The AgNPs exhibited enhanced antioxidant activity, suggesting their use in various applications	[45]
18	Antioxidant, hypoglycemic, and cytotoxic activities	*Gracilaria lemaneiformis*	Selenium	The nanoconjugates exhibited significant antioxidant, hypoglycemic, and cytotoxic activities, along with low hemolytic properties, demonstrating potential as an antioxidant supplement and a possible medication in diabetes	[54]
19	Anticancer and antimicrobial activity	*Gracilaria foliifera*	Gold	The AuNPs exhibited anticancer effects and antimicrobial activity, suggesting their application as promising agent for these purposes	[53]
20	Immunomodulatory, antioxidant, and antitumoral activity	*Chondrus crispus*, *Gelidium corneum*, and *Porphyra linearis*	Gold	Antioxidant and immunomodulatory properties of AuNPs were observed, where *P. linearis*-derived AuNPs exhibited the best antioxidant capacity, suggesting their potential applications in immunotherapy	[44]
21	Antibacterial activity	*Kappaphycus alvarezii*	Silver	A good antibacterial activity of AgNPs against various *E. coli* strains was observed, indicating their potential as safe and effective alternative to antibiotics for preventing bacterial infections	[34]
22	Antibacterial activity	*Halymenia porphyriformis* and *Solieria robusta*	Silver	These AgNPs exhibited moderate antibacterial activity and may serve as potential agents for the treatment of oral bacterial pathogens	[56]
23	Antioxidant activity	*Jania rubens*	Chitosan-tripolyphosphate	These NPs demonstrated strong antioxidant activity and effective radical elimination	[58]
24	Antibacterial, antioxidant and anticancer activities	*Hypnea valentiae*	Silver	Activity of the AgNPs was observed on all the evaluated biological tests, demonstrating a strong capacity to eliminate free radicals, action against four pathogens, and effectiveness against human colon and lung cancer cells	[31]
25	Antioxidant, antimicrobial, and anticarcinogenic activity	*Halymenia venusta*	Gold	Antioxidant, antimicrobial, and anticancer activities of the AuNPs were obtained, highlighting the inhibition of free radicals and lung cancer cells, demonstrating significant potential for this purpose	[57]
26	Anticancer activity	*Kappaphycus alvarezii*	Graphene oxide	A new multifunctional nanohybrid system for drug delivery was developed, demonstrating significant cytotoxic activity against cancer cells, suggesting a potential as biocompatible nanomaterial and a model for anticancer drugs	[36]
27	Antibacterial activity	*Gelidiella acerosa*	Gold	Tests demonstrated antibacterial activity of these AuNPs against *S. aureus*, indicating potential for biomedical applications	[47]
28	Treatment of risk factors associated with kidney injury	*Gracilaria oblongata*	Titanium dioxide	Extracts of *G. oblongata* protect against acute kidney injury by TiO2 NPs by reducing oxidative stress and inflammation	[55]
29	Antioxidant and anticancer activity	*Hypnea valentiae*	Gold	These AuNPs exhibited strong antioxidant activity and potential anticancer effects, proving capability in eliminating free radicals and inhibiting the growth of lung cancer cells	[32]
30	Antioxidant and anticarcinogenic activity	*Champia parvula*	Gold	Significant antioxidant activity and anticancer effects of these AuNPs against lung cancer cells were observed, indicating potential for the development of new anticancer drugs	[37]
31	Antioxidant and antitumor potential	*Hypnea valentiae*	Iron oxide	These IONPs demonstrated significant antioxidant activity and anticancer efficacy, particularly against breast cancer cells, suggesting their potential for future targeted treatments for this cancer	[43]
32	Anticarcinogenic activity	*Porphyridium purpureum*	Silver and zinc oxide	The nanocomposite exhibited strong cytotoxicity against breast cancer cells, highlighting its significant potential for anticancer therapies	[60]
33	Antimicrobial activity	*Kappaphycus alvarezii*	Silver	Effective antimicrobial action of these AgNPs was observed, indicating their potential for applications for food preservation and biomedical products	[35]
34	Antibacterial activity	*Gracilaria fisheri*	Gold and silver	Significant antibacterial activity against shrimp pathogens of NPs was observed. Additionally, these NPs improved the survival rate of infected shrimps, suggesting their potential as a promising agent to fight against *Vibrio* sp. Infections	[52]
35	Anticoagulant properties	*Acanthophora* sp.	Copper	These CuNPs exhibited a strong anticoagulant effect, suggesting their potential as anticoagulant	[42]
36	Antimicrobial activity	*Gracilaria verrucosa*	Copper oxide	A limited antimicrobial activity of these CuONPs was observed, inhibiting only *E. coli* at the highest concentrations tested, indicating the need for further research on antimicrobial potential of these NPs	[40]
37	Larvicidal and neurotoxicity activity	*Gracilaria corticata*	Silver	These AgNPs showed efficacy in killing the larvae of *A. aegypti*, *A. stephensi*, and *C. quinquefasciatus*, indicating their potential to be used as nano larvicidal agent	[49]
38	Drug deliver for cancer therapy	κ-carrageenan (commercial)	Copper oxide	The CuO based NPs exhibited strong cytotoxicity and effective paclitaxel release, with metabolic alterations, and inhibited cell proliferation linked to apoptosis from reactive oxygen species. It also reduced mitochondrial membrane potential, showcasing a novel targeted drug delivery approach	[65]
39	Antioxidant, antimicrobial e anticancer activity	*Champia parvula*	Silver	Significant antioxidant, antimicrobial and anticancer activities of these AgNPs were observed, with high efficacy against lung cancer cells and the control of bacterial and fungal pathogens. Therefore, they show promise for the development of new nanomedicines	[38]

### 2.3. General Industry

In the general industry field, eight articles were found, published in 2018 (n = 1), 2022 (n = 3), 2023 (n = 3), and 2024 (n = 1). These studies included the following red algae for the development of novel NPs: *Palmaria palmata* (n = 1), *Kappaphycus alvarezii* (n = 2), *Gracilaria canaliculata* (n = 1), *Pterocladia capillacea* (n = 1), *Gelidiella acerosa* (n = 1), and κ-Carrageenan (commercial; n = 2), which was sourced from red algae. Regarding the research aims, the focus was on optimizing methodologies (n = 2), packaging (n = 2), detection and rapid removal of Hg^2+^ ions (n = 1), potential to absorb Ismate Violet 2R (IV2R) ions (n = 1), multiple industrial applications (n = 1), and photocatalytic degradation of commercially available dyes, methylene blue and rhodamine B (n = 1) (Table 2).

Among papers reporting on the algae *P. palmata*, López-Mayán et al. [68] described an optimized method for separating silver NPs (AgNPs) from the algae using an enzyme-assisted hydrolysis method combined with ultrasounds. After exposing *P. palmata* to AgNPs, an isolation test was conducted between these matrices. Briefly, it was found that the type of sonication (bath vs. ultrasonic probe), ultrasound amplitude, sonication time, sonication mode (pulsed vs. continuous), enzyme mixture concentration, and enzyme hydrolysis time were parameters significantly affecting the process. After a fine optimization of the process, good repeatability, sensitivity, and analytical recovery were observed. The method was validated and applied to ten different algal samples (*P. palmata* and the green seaweed *Ulva* sp.) to study the accumulation mechanism of the nanomaterial by these organisms. The initial concentration of silver detected in unexposed seaweeds was low (0.010–0.021 μg/g), suggesting that these organisms did not accumulate significant amounts of the metal before exposure. However, when exposed to AgNPs, a significantly higher concentration was observed in algal tissues, especially in *P. palmata* (0.34 to 0.72 μg/g), indicating its bioaccumulation capacity. The average size of AgNPs was similar among the algae, ranging from 20 to 22 nm. The results suggested that red seaweeds present the potential to accumulate AgNPs, with implications for ecotoxicology and environmental safety.

Sudhakar, Venkatnarayan, & Dharani [69] described *K. alvarezii* use for the preparation of bio-nanocomposite films for different applications as an alternative to common refined biopolymers, such as κ-carrageenan. For this purpose, mixtures of algal extracts with metal oxide NPs, such as zinc oxide (ZnONPs), cupric oxide (CuONPs), and silicon dioxide (SiO_2_NPs), were prepared. The incorporation of NPs, K. alvarezii (KBF), and κ-carrageenan (CBF) into the bioplastic film matrices resulted in specific surface morphology, with increased roughness and reduced UV transmittance, as well as reduced water absorption rate, moisture content, and solubility, compared to controls. This is due to nanoparticles that, when combined with the biopolymer matrix, act as fillers and form stable hydrogen bonds with the macromolecular structure, filling up spaces that are usually occupied by water molecules. As a result, NPs provide structural rigidity and, actually, a moisture barrier. However, a difference was observed in the film average roughness, with the KBF nanocomposite showing a higher roughness compared to CBF, while the latter showed an improved tensile strength. The CBF film was clear and transparent, with high transmittance (50.3% at 660 nm), while the KBF film had a light brown color, with a transmittance of 30.6% at 660 nm. The thickness of the samples varied from 92 to 122 μm. After the structural characterization, the films were tested for antibacterial activity against *Staphylococcus aureus* and *Escherichia coli* (using 200 mg of films). Both samples exhibited strong antibacterial activity, with KBF showing a better performance compared to CBF. The observed antimicrobial effect was proposed as novel technology to delay the degradation of bio-nanocomposite films. Therefore, *K. alvarezii* could be applied in various industrial applications, such as in the food industry as a convenient biopolymer alternative for the fabrication of hydrogels, scaffolds, and graft material manufacturing.

Additionally, the aforementioned inorganic nanomaterials, cinnamon NPs have a potential as well in enhancing the performances of biopolymer films made from *K. alvarezii*. Indeed, the study by Rizal et al. [70] explored the effect of NPs when incorporated in the films at different concentrations (1, 3, 5, and 7% *w*/*w*). The results showed that the addition of cinnamon NPs improved the morphological, mechanical, thermal, wettability, and antibacterial properties of the nanocomposite films. The hydrophobicity of the film increased by increasing the NP concentration. Tensile and thermal properties were significantly enhanced by the incorporation of cinnamon NPs into the algal film. In addition, the films exhibited antimicrobial activity against *E. coli* (inhibition zone of 11.39 mm), *S. aureus* (10.27 mm), and Salmonella (12.46 mm). Thus, the *K. alvarezii* film reinforced with cinnamon NPs could be positively employed for packaging applications.

In the research carried out by Parsaee [71], gold NPs (Au-NPs) were synthesized using the extract of the red seaweed *Gracilaria canaliculata* as a reducing and stabilizing agent. Subsequently, these NPs were tested as a chemiosensor for the rapid detection and removal of mercury ions (Hg^2+^). The Au-NPs were produced using an ultrasound-algal-assisted synthesis and then applied to a silica membrane with the dye rhodamine B (RhB) by electrospinning technique. The membrane was fixed onto a glass slide and used to detect Hg^2+^, giving an RhB color change upon contact with mercury and leading to a sensitive detection. Moreover, the sensor could remove up to 99% Hg^2+^ from water and was able to detect mercury concentrations in the nanomolar range (i.e., 2.21 nM by a colorimetric method and 1.10 nM by a fluorescent method). In addition, the authors emphasized that the sensor could be reused more than 10 times with high efficiency.

The production of green zinc oxide NPs (ZnO-NPs) derived from *Pterocladia capillacea* and the evaluation of the ability to absorb Ismate Violet 2R (IV2R) ions from an aqueous solution were conducted by Mansour et al. [72]. The green synthesis led to ZnO-NPs with an average pore size of 2.5 nm. The maximal removal efficiency of IV2R reached 99% when the incubation parameters were optimized, namely ZnO-NPs concentration, environment pH, temperature, and contact time. The maximum adsorption capacity of the ZnO-NPs for the dye resulted in 72.24 mg g^−1^. The study suggested that the formulation could be promising for water treatments.

Among studies involving the use of commercial κ-carrageenan (obtained from red seaweeds), Heydari, Bakhtiarian, and Khodaei [73] proposed the preparation and catalytic application of a magnetic nanocatalyst based on κ-carrageenan. The κ-carrageenan was modified with metformin (Met) and subsequently used to coat magnetite (Fe_3_O_4_) nanoparticles. The resulting NPs exhibited an average size of 23 nm. These NPs demonstrated excellent activity for the chemical synthesis of dihydropyrano[2,3-c]pyrazoles, achieving yields of 88–94%. According to the authors, the synergistic effect of sulfonic acid groups and amino groups might be responsible for the catalytic activity of these NPs. Thus, a reusable, cost-effective, and highly active biobased catalyst was obtained, which could be applied for most acid-catalyzed organic reactions.

Kumari et al. [74] described the application of commercial κ-carrageenan as a matrix to create a nanocomposite film containing copper (Cu) NPs, which were previously nucleated with an *Argemone mexicana* herb extract. The NPs exhibited an estimated size of 28 nm and, when combined in the κ-carrageenan matrix, led to improved thermal stability, elastic properties, resistance to water vapor, and UV resistance compared to the pure Cu NP film. In addition, the copper-containing nanocomposite film showed strong antimicrobial properties against *S. aureus* and *E. coli*. Lastly, grapes (for 12 days) and cottage cheese (for 7 days) were well-preserved using this film, and the quality of the food was maintained without any additional care. This evidence suggests that the new organometallic film might act as an efficient and environmentally acceptable alternative for food packaging.

Subbulakshmi et al. [47] developed gold NPs using an extract from *Gelidiella acerosa*. Authors obtained spherical NPs ranging from 5 to 20 nm in size, which were tested for the photocatalytic degradation of commercially important dyes, such as methylene blue and rhodamine B. Several amounts of NPs were mixed with the dyes for subsequent analysis of their photocatalytic degradation. After exposure to sunlight under continuous stirring, a decrease in the dye UV-visible peak was observed and interpreted as an index of degradation of the exposed compound. The degradation fraction catalyzed by gold NPs was 83.21% for methylene blue and 88.36% for rhodamine B, underlining the potential of *G. acerosa*-derived gold NPs for environmental applications.

**Table 2:** Published articles in the PubMed and Web of Science databases (2014–2024) dealing with nanoparticles based on red seaweeds for applications in general industry.

Nº	Target Effect	Species Used	Core Nanoparticle	Results/Potential	References
1	Detection and rapid removal of Hg^2+^ ions	*Gracilaria canaliculata*	Gold	Newly synthesized Gold NPs were applied in a glass probe chemiosensor, demonstrating the effective detection and removal of mercury ions from water, with high sensitivity and reusability	[71]
2	Potential to absorb Ismate violet 2R ions	*Pterocladia capillacea*	Zinc oxide	NPs were developed as highly effective for removing Ismate Violet 2R dye from aqueous solutions, showing a potential for water treatment applications	[72]
3	Methodology optimization	*Palmaria palmata*	Silver	The algae demonstrated significant capacity to accumulate silver NPs, indicating its potential for removing environmentally dangerous nanomaterials	[68]
4	Multiple industrial applications	*Kappaphycus alvarezii*	Zinc oxide, cupric oxide, and silicon dioxide	Bio-nanocomposite films with enhanced antibacterial properties and modified physical characteristics were developed, suggesting applications in food industry	[69]
5	Methodology optimization	κ-Carrageenan (commercial)	Magnetite	NPs with high catalytic activity for synthesizing dihydropyrano[2,3-c]pyrazoles were proposed, suggesting applications as a reusable and cost-effective biobased catalyst for acid-catalyzed organic reactions	[73]
6	Packaging	*Kappaphycus alvarezii*	Cinnamon	Improved mechanical, thermal, and antibacterial properties of novel nanofilms were demonstrated for food packaging applications	[70]
7	Photocatalytic degradation	*Gelidiella acerosa*	Gold	Degradation of commercially important dyes was obtained, suggesting environmental applications	[47]
8	Packaging	κ-Carrageenan (commercial)	Copper	Nanocomposite films exhibiting enhanced thermal stability, elasticity, and antimicrobial properties were proposed as promising, and environment-friendly alternative for food packaging	[74]

### 2.4. Agriculture

In the agricultural context, only three scientific papers were identified regarding the synthesis and application of nanoparticles (NPs) based on red algae, published in 2019, 2021, and 2023. These studies focused on disease control (n = 3) and growth support (n = 2). The total count of subjects exceeds the number of papers because two of the articles address more than one topic (Table 3).

The study by Roseline et al. [75] reports on the green synthesis of NPs by using aqueous extracts from four red algae (*Gracilaria corticata*, *G. edulis*, *Hypnea musciformis,* and *Spyridia hypnoides*) and tested their effects against agricultural phytopathogens, including the bacterium *Xanthomonas axonopodis* pv. *Citri*, causing citrus cancer, and *X. oryzae* pv. *oryzae*, responsible for rice blast, as well as the fungus *Ustilaginoidea virens*, also a cause of blast. The aqueous extracts of the algae were mixed with silver nitrate in order to prepare coated silver nanoparticles (AgNPs), where the presence of proteins and sulfated polysaccharides (agar and carrageenan) functional groups on the NPs was observed. The functionalized AgNPs exhibited a spherical shape with an average size of 37 nm (*G. corticata*), 54 nm (*G. edulis*), 53 nm (*H. musciformis*), and 49 nm (*S. hypnoides*). Noteworthy, the antibacterial activity was higher with respect to the antifungal activity, and, among bacteria tested, the inhibitory effect was highest against *Xanthomonas axonopodis*, revealing a specificity toward the examined microorganisms.

Moreover, these RNPs were demonstrated to be a suitable component for the formulation of nanopesticides. The subsequent research by Roseline, Sudhakar & Kulanthaiyesu [76] demonstrated the feasibility of the synthesis of silver nanoparticle composites (AgNPCs) using the extract from the red alga *Calliblepharis fimbriata*. The nanocomposites exhibited a spherical morphology with a size ranging from 25 to 30 nm. After a preliminary characterization, the effect of NPs on the germination of *Oryza sativa* seeds was evaluated, as well as their action against the bacterium *X. oryzae* pv. *oryzae* (which causes the rice blight disease) and on the stimulation of the growth of rice plants infected by bacterial leaf streak. It was found that the NP formulation stimulated plant growth, especially when used at 50 μg/mL. In addition to promoting seed germination, increases in root length and shoot length were observed, along with higher fresh and dry weight in treated plants. Regarding the antibacterial effect, it was noted that, at the same concentration (i.e., 50 μg/mL), a reduction of bacterial colonies in rice seedlings after 10 days from inoculation was observed. In addition, at the same concentration, AgNPCs also promoted the growth of rice plants and induced an increase in the content of photosynthetic pigments.

Finally, another study from the same group [77] highlighted the use of aqueous extracts from *Gracilaria crassa* and *Grateloupia litophila* for the green synthesis of silver NPs. The as-obtained AgNPs exhibited an average size of 51 nm (*G. crassa*) and 21 nm (*G. litophila*), and these were tested against two agricultural pathogens, *X. axonopodis* pv. *citri* and X. *oryzae* pv. *oryzae*, and their effects on seed germination and on the growth of rice seedlings were analyzed. Regarding antibacterial activity, it was found that the AgNPs by *G. litophila* exhibited a higher antibacterial activity compared to *G. crassa*-derived NPs. The activity increased with AgNP concentrations (10 and 20 µg/mL), suggesting a selective antibacterial behavior. Concerning their effect on seed germination, 50 and 100 μg/mL concentrations were tested. Compared to controls, 50 μg/mL of both AgNP sizes (51 and 21 nm) resulted in significantly higher germination, germination index, root and shoot growth, and fresh and dry weight.

## 3. Discussion

Overall, the pharmaceutical/medical sector accounted for the largest number of manuscripts (n = 39), indicating a comprehensive exploration of the use of natural products derived from red algae. A notable diversity of biological activities was investigated, with a particular emphasis on anticancer activity, which emerged as the most studied area (21 mentions). This focus can be attributed to the rising incidence of cancer and the global demand for effective treatments [78,79]. Research on anticancer agents derived from natural products has shown considerable promise, given the potential of these compounds to inhibit tumor growth and mitigate the side effects commonly associated with synthetic chemotherapy [80,81]. Conversely, other biological activities, such as neurotoxicity and prediction of kidney injury, have received relatively less attention, likely due to the complexity and specificity of the associated pathologies.

Clinical implications of studied biological activities of nanomaterials are wide-ranging and have the potential to directly affect the development of new medications and treatments [82,83]. The aforementioned findings from anticancer and antimicrobial activities, for example, could lead to the formulation of innovative therapies utilizing natural compounds in combination with a nanotechnological approach. Indeed, the synergistic roles arising from the interaction between seaweed metabolites and nanoparticles leading to a single product may not only enhance treatment efficacy but also provide a holistic and less toxic approach compared to conventional therapies [84]. Furthermore, the use of seaweed extracts, which already demonstrated beneficial activities, may open new avenues for developing novel drugs with unique and multifunctional properties [84,85]. Thus, the findings presented in reported studies not only encourage further research into the application of natural products but also highlight the importance of exploring less-studied biological activities. The understanding of these aspects could be crucial for developing new medical treatments, contributing to the advancement of pharmacology and modern medicine.

In addition to the pharmaceutical sector, two other areas emerged: general industry and agriculture, which had significantly lower numbers of articles, representing only 16% and 6%, respectively. The analysis of articles focused on developing natural products for practical applications across various industries, particularly in food packaging and environmental remediation, highlights a diverse range of algae species utilized. This diversity suggests that each type of algae can offer unique properties, thereby expanding the possibilities for innovation. In this vision, it would be worthy to carry out similar analyses considering other species of red seaweed. Additionally, these novel green protocols for synthesizing NPs able to remove heavy metals and contaminants, such as mercury, underscore the ecological relevance of this research. The utilization of low-cost biological resources, such as seaweeds, to develop sustainable solutions not only contributes to mitigating environmental impacts [86] but also promotes responsible industrial practices [70]. Thus, the production and application of NPs derived from red algae could represent a significant advancement toward a sustainable future, with multiple benefits for both industry and the environment.

It is worth mentioning that despite the limited number of publications on red seaweeds found in the current study, green-synthesized NPs from several natural sources are widely applicable in agriculture. Indeed, NPs were proposed as nanofertilizers or plant growth stimulants, nanopesticides, carriers for conventional pesticides, and antibacterial agents [87]. Albeit being at an embryonal stage, the employment of red seaweed NP in this field envisages possible future developments.

Overall, in view of actual applicability, it is important to consider NP phytotoxicity, especially concerning overdosing. Therefore, experiments testing different dosages, as well as the potential soil and/or plant toxicity, are essential to ensure their safe usage in agriculture. That said, it is surprising to evince that only a restricted number of papers about green-synthesized NPs from red algae were reported, particularly when compared to the plethora of biomedical publications. This is even more unexpected considering the magnitude of the agricultural sector, demanding feasible and highly scalable innovative solutions. In this view, as demonstrated by the studies by Roseline et al. [75,76,77], nanomaterials derived from different red seaweeds can be elective candidates for agricultural applications. Thus, aiming at enhancing crop productivity and expanding the choice of novel and safer alternatives to conventional pesticides, red algae nanomaterials merit significantly more effort in terms of in-depth studies on their use for agricultural purposes.

### Gaps and Prospects

In light of the increasing demand for sustainable and effective solutions in agriculture, industry, and medicine, the present study focuses on the properties and applications of nanoparticles derived from red algae. By analyzing their efficacy and potential for innovation across various industrial sectors, reported papers highlighted the unique characteristics of these naturally derived nanomaterials. The growing interest in the beneficial properties of seaweed not only underscores their relevance in addressing contemporary challenges but also opens up numerous opportunities for the development of new products. This exploration aims at contributing to the advancement of knowledge in the field while meeting the evolving demands of the market. However, some key points of relevance need to be addressed. First, the still limited number of studies in general industry and agriculture areas indicate a significant opportunity for future research, which could finally promote the translation of actual novel technological solutions from the laboratories to real-world scenarios. In particular, an important goal would be if algal-derived nanoparticles could replace the harmful products currently employed in the agricultural sector with cleaner and safer alternatives for both humans and the environment [88].

As far as the pharmaceutical/medical field is concerned, the topic most represented herein, an in-depth research on the mechanisms of action of nanoparticles derived from red algae, would be highly required. It is well-known that surface plays a main role in the NP interplay with biological systems. Biomolecular shells (bio-coronas) endow abiotic cores with a biological identity that dictates their fate in biological systems and therefore their application potential in biomedicine [89,90,91].

**Figure 3:**
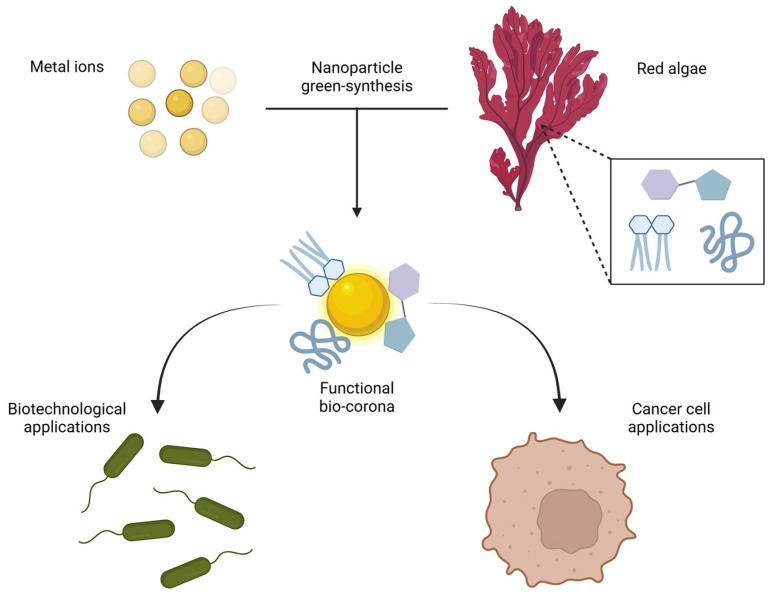
Schematic representation of NP green synthesis using red seaweeds. Algal extracts are rich in primary metabolites (amino acids, sugars, fatty acids, polysaccharides, organic acids, proteins, fibers, vitamins, and biogenic amines), and secondary metabolites (phenolic compounds and pigments) can compete in the bio-corona development. The latter composition influences the fate of NPs in biological systems and, therefore, their feasibility as therapeutics. The image was produced with Biorender with “Created in BioRender. Rilievo, G. (2025) https://BioRender.com/8b2cmu2 (accessed on 10 March 2025)”.

Thus, it is very likely to assume that red seaweeds, due to the richness of primary and secondary metabolites, likely determine a kaleidoscope of multi-component bio-coronas and biological actions. In fact, the use of algal extracts as reducing/capping agents or surface modifiers in NP green synthesis would plausibly involve the competition of a variety of biomolecules in the formation of bio-coronas (Figure 3). This vision is fully in harmony with the number of specific nano-bio interactions (e.g., with prokaryotic or eukaryotic cancer cells) that emerged from the analysis of the literature encompassed by the present study.

A better understanding of the correlation between the bio-corona compositions and the mechanisms of action of these algal-derived nanomaterials could significantly enhance their application in various fields, such as medicine and pharmacology. Indeed, the punctual characterization of the aforementioned bio-coronas, along with an elucidation of interactions between these nanoparticles and biological systems, will be essential for the optimization of their effectiveness and for the targeting of specific pathways, ultimately leading to efficient therapeutic strategies and innovative healthcare solutions [92,93]. Furthermore, the large-scale production of nanoparticles continues to face significant technical and economic challenges, such as scalability, green-economy standards, and costs. The overcoming of these obstacles is crucial for the commercialization of nanoparticles derived from red algae. Thus, studies focusing on the scalability and economic feasibility of NPs synthetic processes are essential to bridge the gap between laboratory research and industrial application. Indeed, the potential for integrating these nanoparticles across various industries can be realized only by developing cost-effective production methods and optimizing synthetic processes, paving the way for sustainable innovations [94,95]. Moreover, the assessment of toxicity and long-term safety of nanoparticles in humans and in the environment remains a critical area of research. Long-term studies are necessary to evaluate potential adverse effects and ensure the safe use of these nanomaterials. As the application of red algae-derived nanoparticles tends to expand, the understanding of their long-term impacts will be vital for regulatory compliance and public acceptance, ultimately contributing to their successful integration into therapeutic and industrial settings [96,97,98].

Finally, it is important to consider that the establishment of guidelines and regulations for the use of nanoparticles across various sectors is also a crucial factor. As the applications of NPs expand, clear regulatory frameworks are necessary to ensure safety and efficacy. The development of proper standards will not only facilitate the responsible use of nanoparticles but also promote public trust and acceptance. Collaborative efforts between researchers, industry stakeholders, and regulatory bodies will be essential for creating comprehensive guidelines that address potential risks while fostering innovation and sustainability in the field [99].

For summarizing, while red algae-derived nanoparticles present promising potential across various fields, including medicine, agriculture, and industry, some significant challenges remain to be addressed. Ongoing research into their mechanisms of action, large-scale production, long-term safety, and regulatory frameworks will be crucial for their successful integration into practical applications. By overcoming these obstacles and fostering collaboration between academia, industry, and regulatory bodies, the full potential of seaweed-synthesized nanoparticles can be realized, paving the way for innovative, sustainable solutions that benefit both human health and the environment.

## 4. Material and Methods

### 4.1. Study Design

The present systematic review was conducted following the PRISMA criteria (Preferred Reporting Items for Systematic Reviews and Meta-Analyses (https://www.prisma-statement.org/prisma-2020-checklist, accessed on 20 September 2024) [100] using two databases, PubMed and Web of Science. The descriptors used were the terms “red seaweed” AND “nanoparticles”, considering publications of scientific articles from the period of 2014 until 30 September 2024.

### 4.2. Quality Assessment e Data Extraction

Only peer-reviewed articles within the established period (2014–2024) were selected. The data were fully extracted from PubMed and Web of Science for further analysis, regardless of whether they would be considered in the results. The data were tabulated into a Microsoft Excel^®^ data sheet (.xlsx), including the title of the paper, the year of publication, the area involved in the study, and the aim of the study.

### 4.3. Inclusion/Exclusion Criteria

**Figure 4:**
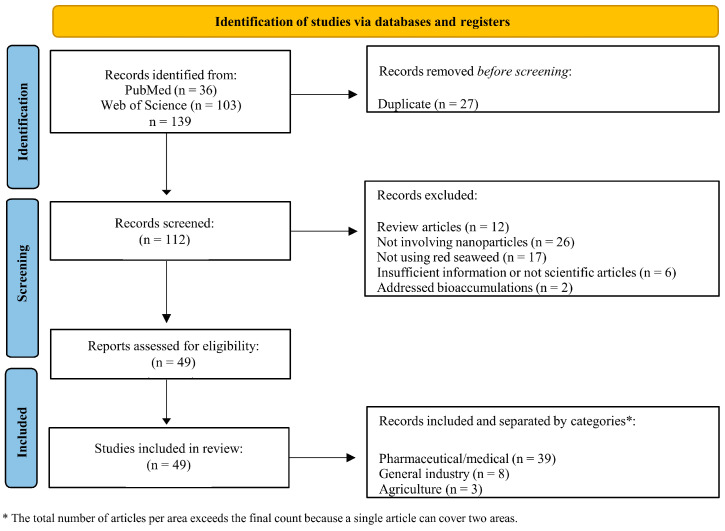
Flowchart based on the PRISMA model with the results of the search carried out in the PubMed and Web of Science databases (2014–2024) using the descriptors “red seaweed” and “nanoparticles”.

After compiling the results from the PubMed (n = 36) and Web of Science (n = 103) databases into the Microsoft Excel^®^ data sheet (.xlsx), duplicate papers were found between the two platforms (n = 27), and copies were excluded. Thus, 112 papers were selected, among which 12 review papers were identified, 26 papers did not address studies involving nanoparticles, 17 papers did not use red seaweed, 6 papers did not provide adequate information or were not scientific articles, and 2 addressed bioaccumulations. A total of 49 papers were identified based on the descriptors used. After a thorough analysis of the manuscripts, they were categorized into three fields: pharmaceutical/medical (n = 39), general industry applications (n = 8), and agriculture (n = 3). The total count (n = 50) exceeds the number of papers (n = 49) because one of the articles addresses two fields (Figure 4).

## 5. Conclusions

This scoping review has revealed that there is a wide range of studies that have explored red weeds as sources of novel NPs, both as surface modifiers or, in particular, starting materials (simultaneously reducing and capping agents). A total of 49 studies published between 2014 and 2024 were identified and categorized into three main sectors: pharmaceutical/medical, general industry, and agriculture fields. Notably, 79% of the research was focused on the pharmaceutical/medical sector, 16% on general industry, and only 6% on agriculture. These findings highlight the dominance of pharmaceutical and medical uses but also underscore the need for increasing the research in agriculture and other underexplored areas.

The high concentration of studies in the pharmaceutical and medical fields suggests that red algae-derived nanoparticles may offer significant potential for the development of therapeutic innovations, especially for anticancer, antimicrobial, and antioxidant properties. This focus reflects the growing demand for biocompatible materials for health-related applications. However, the lower fraction of studies on agriculture (6%) points to an untapped potential for these nanoparticles to contribute to areas such as crop protection, soil enhancement, and sustainable agricultural practices. Given the global challenges of food security and environmental sustainability, further exploration in this field could yield valuable solutions. Moreover, the versatility of red algae-derived nanoparticles in general industry, comprising 16% of the studies, shows their potential for broader technological applications, including environmentally friendly manufacturing processes and materials. Their biocompatibility and eco-friendly production methods align well with the increasing emphasis on sustainable industrial practices. This comprehensive study contributes to the awareness that a constant and punctual update of the state of the art is of primary importance for taking full advantage of natural materials.

## Figures and Tables

**Table 3:** Published articles in the PubMed and Web of Science databases (2014–2024) dealing with nanoparticles with red seaweed components for agricultural applications.

Nº	Target Effect	Species Used	Core Nanoparticle	Results/Potential	References
1	Disease or pest control	*Gracilaria corticata*, *Gracilaria edulis*, *Hypnea musciformis*, and *Spyridia hypnoides*	Silver	Green synthesis of NPs with anti-phytopathogen activity as suitable resource for the formulation of nanopesticides	[75]
2	Control plant disease and support plant growth	*Calliblepharis fimbriata*		NP composites stimulate seed germination and are effective against agricultural bacteria, which can enhance the vegetative growth of rice	[76]
3		*Gracilaria crassa* and *Grateloupia lithophila*		Green-synthesized NPs produced for controlling bacterial diseases in plants and for supporting plant growth	[77]

## Data Availability

The data presented in this study are available upon request to the corresponding author due to privacy and amount of data generated.
